# The Impact of Uremic Toxins on Vascular Smooth Muscle Cell Function

**DOI:** 10.3390/toxins10060218

**Published:** 2018-05-29

**Authors:** Lucie Hénaut, Aurélien Mary, Jean-Marc Chillon, Saïd Kamel, Ziad A. Massy

**Affiliations:** 1 MP3CV, EA7517, CURS, Jules Verne University of Picardie, 80054 Amiens, France; aurelien.mary@u-picardie.fr (A.M.); jean-marc.chillon@u-picardie.fr (J.-M.C.); said.kamel@u-picardie.fr (S.K.); 2 Department of Pharmacy, Amiens University Medical Center, 80000 Amiens, France; 3 DRCI, Amiens University Hospital, 80054 Amiens, France; 4 Laboratory of Biochemistry, Amiens University Hospital, 80054 Amiens, France; 5 Ambroise Paré University Hospital, Division of Nephrology, APHP, 92100 Boulogne-Billancourt/Paris, France; ziad.massy@aphp.fr; 6 INSERM U1018, Team 5, CESP, UVSQ, Paris-Saclay University, 94800 Villejuif, France

**Keywords:** chronic kidney disease, uremic toxins, vascular smooth muscle cells

## Abstract

Chronic kidney disease (CKD) is associated with profound vascular remodeling, which accelerates the progression of cardiovascular disease. This remodeling is characterized by intimal hyperplasia, accelerated atherosclerosis, excessive vascular calcification, and vascular stiffness. Vascular smooth muscle cell (VSMC) dysfunction has a key role in the remodeling process. Under uremic conditions, VSMCs can switch from a contractile phenotype to a synthetic phenotype, and undergo abnormal proliferation, migration, senescence, apoptosis, and calcification. A growing body of data from experiments in vitro and animal models suggests that uremic toxins (such as inorganic phosphate, indoxyl sulfate and advanced-glycation end products) may directly impact the VSMCs’ physiological functions. Chronic, low-grade inflammation and oxidative stress—hallmarks of CKD—are also strong inducers of VSMC dysfunction. Here, we review current knowledge about the impact of uremic toxins on VSMC function in CKD, and the consequences for pathological vascular remodeling.

## 1. Introduction

Vascular smooth muscle cells (VSMCs) are the cellular components of the medial layer of normal vessel walls. The VSMCs’ primary role is to control the diameter of the lumen by contracting and relaxing dynamically in response to vasoactive stimuli, which enables the maintenance of an appropriate blood pressure [1]. In healthy young blood vessels, the VSMCs mainly display a contractile phenotype, and blood pressure is well regulated. However, in response to various stimuli (including mechanical forces, growth factors, pro-inflammatory cytokines and vascular injury), VSMCs can reprogram their gene expression patterns and take on other functions. This considerable phenotypic plasticity enables VSMCs to adapt to their microenvironment. In particular, VSMCs can switch from a contractile phenotype (characterized by the expression of smooth muscle alpha actin, smooth muscle myosin heavy chain (SMMHC), SM22α and calponin [2] to a synthetic phenotype. In this case, the VSMCs change their morphology, proliferate, become migratory, and synthesize large amounts of extracellular matrix (ECM) components-leading to arterial remodeling. Even though these functional changes are essential for the repair of vascular damage [3], they can also favor the development of various vascular diseases, including atherosclerosis, calcification and hypertension. These conditions reduce heart perfusion, impair left ventricular function, and increase the risk of developing cardiovascular disease (CVD).

**Figure 1:**
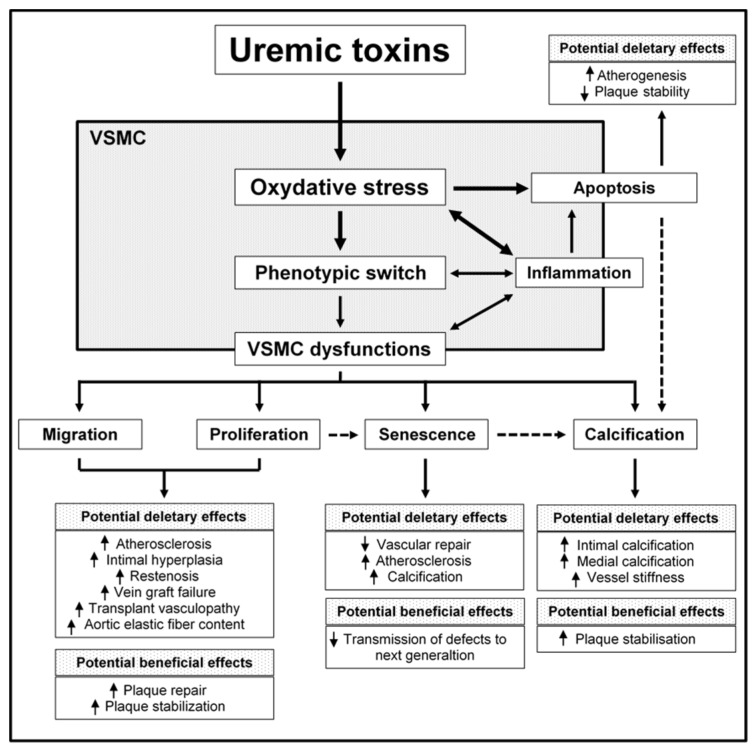
A schematic view of the impact of uremic toxins on VSMC cell function and then vascular function. VSMC: vascular smooth muscle cell.

Cardiovascular mortality and morbidity rates are higher in patients suffering from chronic kidney disease (CKD) than in the general population [4]. Traditional risk factors for cardiovascular events (such as hypertension, diabetes, inflammation, and dyslipidemia) contribute to the pathogenesis of CKD-induced CVD. Furthermore, CVD in CKD is associated with several non-traditional risk factors, including CKD-associated disorders of bone and mineral metabolism, inflammation, oxidative stress, and the accumulation of uremic toxins. Together with the hemodynamic changes observed in CKD, this altered metabolic state promotes deep vascular wall remodeling with intimal hyperplasia, accelerated atherosclerosis, excessive vascular calcification, and vascular stiffness [5,6]. Under uremic conditions, the expression of markers of a contractile VSMC phenotype (such as calponin, SM22α and SMMHC) is dramatically reduced [7,8,9] and corresponds to a decrease in aortic constriction [8]. This phenomenon is associated with changes in VSMC proliferation, apoptosis, migration, and senescence, and the onset of calcification. Evidence from cell-based and animal models suggests that uremic toxins (which have an important role in CVD morbidity and mortality in CKD patients [10] may be directly responsible for VSMC dysfunction. Here, we review current knowledge about the involvement of uremic toxins (particularly inorganic phosphate (Pi), tumor necrosis factor α (TNF-α), interleukin-6 (IL-6), indoxyl-sulfate (IS) and advanced glycation end-products (AGEs)) in CKD-induced VSMC dysfunction, and the consequences for vascular remodeling. The effects of fibroblast growth factor 23 FGF-23 and paracresyl sulfate (PCS) are also discussed, when documented. Figure 1 provides a schematic view of the impact of uremic toxins on VSMC cell function and then vascular function. Table 1 summarizes the mechanisms by which uremic toxins impact VSMC function.

## 2. The Impact of Uremic Toxins on VSMC Function

### 2.1. Proliferation 

Arterial VSMCs exposed in vitro to uremic conditions exhibited the phenotypic changes associated with increased proliferation [7], and uremia increased the medial area in ApoE^−/−^ mice [8]. In the context of atherosclerosis, successful plaque repair requires VSMCs to proliferate and synthesize matrix. Therefore, an increased VSMC count within atherosclerotic lesions is generally associated with increased plaque stability [11]. In contrast, abnormal VSMC proliferation leads to (i) the development of diabetic vascular disease, and (ii) complications of procedures used to treat atherosclerotic diseases (e.g., postangioplasty restenosis, vein graft failure, and transplant vasculopathy). Furthermore, the excessive proliferation of medial VSMCs results in a low aortic elastic fiber content—a phenomenon that may be responsible for the increased aortic stiffness and decreased aortic compliance observed in CKD patients [12].

Over the last few decades, a growing body of evidence has suggested that protein-bound uremic toxins contribute significantly to uremia-induced, excessive VSMC proliferation. In vitro, acute exposure to IS promoted the proliferation of rat and human VSMCs [13,14,15,16,17]. This effect was abolished by blockade of the IS transporter organic anion transporter 3 [13]. The production of reactive oxygen species ROS is a critical event in the initiation of IS’s effects on VSMCs. Accordingly, exposure to antioxidants (such as vitamin E, vitamin C, and *N*-acetylcysteine (NAC)) inhibited IS-induced VSMC proliferation [14]. In particular, the IS-induced NADPH-oxidase-dependent production of ROS increased platelet-derived growth factor (PDGF) receptor expression and phosphorylation, which sensitized VSMCs to PDGF-BB-induced proliferation [15]. The proliferative effect of IS had been attributed to various signaling pathways including MAPK P42/44 and P38 [13,15], AhR/NFkB [17] and prorenin/PRR [16]. In rat embryonic aortic A7r5 VSMCs cultured in vitro, acute exposure to IS also favored proliferation after induction of GLUT1 expression through the Akt/TSC2/mTOR/S6K signaling pathway. This effect is associated with increased expression of pro-proliferative cyclin D1 and p21 mRNA and reduction of antiapoptotic p53 mRNA [18]. In contrast, chronic exposure to IS decreased VSMC proliferation. These antiproliferative properties were associated with elevated ROS production and upregulation of the cell cycle inhibitors p21 and p27 [19]. As discussed below, IS also promotes VSMC senescence [20]. Therefore, the low level of in vitro VSMC proliferation associated with long-term IS exposure might reflect a state of replicative senescence that follows the increase in proliferation associated with acute IS exposure. In contrast, despite its pro-oxidative properties, PCS did not modulate VSMC proliferation in vitro [21]. However, in ApoE^−/−^ mice that underwent 5/6 nephrectomy, treatment with PCS increased the proliferation of VSMCs within atherosclerotic lesions [22]. Further studies are therefore required to establish the true impact of PCS on VSMC proliferation [21]. It is noteworthy that PCS increased the phenylephrine-induced contraction in aortic rings cultured in vitro, independently of endothelial cells; this might ultimately lead to inward eutrophic remodeling of the vessels [21]. 

AGEs markedly accumulate in the plasma with gradual decline in glomerular filtration rate and are now considered as uremic toxins. The role of AGE is well known in diabetic vasculopathy, and could be responsible at least for a part of the uremic vasculopathy [23]. Indeed, the interaction between AGEs and the receptor for advanced glycation end-products (RAGE) induced the in vitro proliferation of rat VSMCs in a time- and dose-dependent manner, via increased ROS production [24,25] and subsequent activation of the NFkB and MAPK signaling pathways [24,25,26,27,28]. Furthermore, VSMC proliferation is suppressed in homozygous RAGE-null mice, relative to wild-type littermates [29]. In C57BL/6 mice, the blockade of ligand-RAGE binding (via the administration of soluble RAGE) reduced VSMC proliferation [29]. In Zucker obese diabetic rats, blockade of the RAGE/ligand interaction suppressed VSMC proliferation and neointimal formation after balloon injury [30]. Together, these data suggest that AGE-RAGE axis may impact VSMCs proliferation in a uremic context. However, to the best of our knowledge, no studies have been performed so far to evaluate the impact of AGEs on VSMC proliferation in this setting. Further studies will be needed to investigate how AGEs influence VSMCs proliferation in uremia. 

Furthermore, CKD status is known to favor microinflammation, in particular through the retention of several proinflammatory cytokines, which are now considered as uremic toxins [31]. Some of them are key inducers of VSMC proliferation [32,33,34,35]. Of these, TNF-α displayed mitogenic properties on VSMCs through the direct activation of NFkB [36] and p42/44 [36] signaling pathways. In VSMCs, the TNF-α-induced activation of NFkB depends on Nox1-dependent endosomal ROS production [36], which again highlights the central role of oxidative stress in pathological VSMC proliferation. Furthermore, it was also recently reported that the TNF-α-induced activation of NFkB promotes rat VSMC proliferation by enhancing expression of tumor-necrosis-factor-related apoptosis-inducing ligand (TRAIL) [33]. The application of an anti-IL-6 antibody reduced TNF-α-induced proliferation of human VSMCs in vitro [35], which suggests that at least some of the mitogenic effects displayed by TNF-α are mediated through the VSMCs’ release of IL-6. It is noteworthy that TNF-α also induced the release of IL-6 by endothelial cells, which indirectly promoted VSMC proliferation [37]. In the same manner, IL-1β and TNF-α indirectly induced VSMC proliferation by promoting the release of PDGF from endothelial cells [34,38]. Furthermore, IL-1β also induced PDGF production by VSMCs, which indirectly stimulated their proliferation [34]. Recently, IL-1β has been reported to upregulate the expression of P2Y_2_ receptor (P2Y_2_R) in rat VSMCs cultured in vitro. Activation of the P2Y_2_R by ATP or UTP in rat VSMCs promoted ERK, AKT, PKC, Rac-1 and ROCK2 pathways, and induced VSMC proliferation [32]. This effect was associated with increased expression of RAGE and its ligand HMGB1, which may amplify IL-1β’s mitogenic properties [32]. Despite the extensive bibliography depicting the proliferative impact of TNF-α and/or IL-1β on VSMCs, no studies have been undertaken to date to evaluate whether these toxins also impact VSMC proliferation in uremic conditions. Further studies will be needed to clarify this concern. 

Even though the majority of uremic toxins reportedly promote VSMC proliferation, it must be noted that high Pi concentrations significantly reduced the metabolic activity and proliferation of human VSMCs in vitro [39]. In particular, exposure to high Pi blocked the progression of human VSMCs into the G1/S step in the cell cycle; this effect was associated with decreased expression of cyclin E and cyclin-dependent kinase 2, decreased retinoblastoma protein (pRb) phosphorylation, and increased expression of the cell cycle inhibitors p15, p21, and p27 [40]. As discussed below, the cell cycle arrest induced by hyperphosphatemia may be an early event in VSMC senescence, which has been shown to have a major role in atherosclerosis and medial calcification.

### 2.2. Migration 

The role of VSMC migration in atherosclerosis is still subject to debate. Indeed, VSMC migration may be beneficial because the cells help to stabilize atherosclerotic plaques. However, once within the atherosclerotic lesion, VSMCs may be subjected to increased cell death, senescence or calcification, and may also undergo a switch towards a pro-inflammatory macrophage-like phenotype that may accelerate plaque progression. Therefore, VSMC migration is thought to have a key role in the initiation and progression of atherosclerosis, and greatly contributes to the enhanced neointimal hyperplasia observed in CKD. In ex vivo cell migration assays, aortic VSMCs harvested from mice with CKD migrated significantly further than VSMCs harvested from control mice. Furthermore, animals with CKD had higher serum levels of osteopontin, a factor known to stimulate VSMC migration [41]. It has therefore been hypothesized that the VSMCs’ migratory phenotype may be responsible for both the enhanced neointimal hyperplasia and increased atherosclerosis observed in CKD.

In vitro, the migration of VSMCs isolated from RAGE-null or Tg SM22-DN-RAGE mice was markedly lower than that of cells isolated from WT mice—suggesting that RAGE activation has a key role in VSMC migration [28]. These data were confirmed by the observation that in vitro, RAGE activation by AGEs induced the migration of rat, human and rabbit VSMCs [42,43,44] and was associated with greater expression of matrix metalloproteinases (MMPs) 2 and 9. Phosphorylation of ERK1/2 is required for the RAGE-mediated migration of human VSMCs [44], and the AGE-induced upregulation of K(Ca)3.1 channels has a critical role in this process [45]. Furthermore, the induction of transforming growth factor beta (TGF-β) in response to AGE/RAGE interaction also promoted VSMC migration in vitro [29]. In particular, activation of the ROCK1 branch of the TGF-β pathway contributed to the RAGE-dependent acceleration of atherosclerosis in diabetic ApoE^−/−^ mice [46]. According to recent research, AGE-induced lipocalin 2 expression (via a RAGE-NADPH oxidase-ROS pathway) enhanced the invasive and migratory properties of human VSMCs [47]. In VSMCs isolated from diabetic rats, AGEs, Src kinase and Cav-1 have important roles in RAGE-mediated inflammatory gene expression and cell migration [26]. Again, the RAGE-mediated production of cytokines and growth factors (such as TNF-α [31] and PDGF [48]) is thought to significantly increase AGE-induced migration. Indeed, the TNF-α-induced activation of the NFkB pathway promoted rat VSMC migration in vitro [33,49] via upregulation of invasion-related MMPs 2 and 9 [50]. After TNF-α-induced NFkB activation, the induction of TRAIL and NAD(P)H:quinone oxidoreductase 1 expression is necessary for VSMC migration [33,51]. Furthermore, TNF-α-induced vimentin expression and reorganization (via activation of the TGF-β1/Smad pathway) is an important step in this process [52]. Early activation of MAPK is a crucial event in TNF-α-mediated signal transduction, leading to VSMC migration [53] and induction of the p38-MAPK/CREB/Rac1 pathway by TNF-α-promoted VSMC migration in vitro [54]. These pro-migratory properties partly depend on TNF-α-induced IL-6 production, which favors actin polymerization and tyrosine phosphorylation of focal adhesion kinase and paxillin [55,56]. It is also noteworthy that IL-lβ increased rat VSMC migration and MMP-2 activity [57]; this effect was partly mediated by upregulation of the P2Y_2_R [32]. Despite the extensive bibliography depicting the pro-migratory impact of TNF-α and/or IL-1β on VSMCs, to the best of our knowledge, the impact of AGEs, TNF-α, IL-1β and IL-6 in VSMCs migration had only been described in non-uremic conditions. Additional studies will be needed to confirm these data in CKD.

When considering the protein-bound uremic toxins, IS is known to induce the NADPH oxidase-dependent production of ROS, activate ERK and p38 MAPK, promote the migration of rat VSMCs cultured in vitro, and sensitize cells to PDGF-BB-induced migration [15]. Furthermore, PCS increased VSMCs’ ability to migrate both in vitro and in atherosclerotic lesions in ApoE^−/−^ mice having undergone 5/6 nephrectomy [22]. This effect was associated with increased levels of MMP-2 and MMP-9 activity, and downregulation of TIMP-1 and TIMP-2 expression in aortic samples from PCS-treated mice.

Confirming the key role of hyperphosphatemia in pathological vascular remodeling, the downregulation of miR143 and 145 (in response to high Pi) increased the in vitro expression of key markers of the VSMC synthetic phenotype (Kruppel-like factors (KLFs) 4 and 5 and versican) [39]. This effect was associated with elevated PDGF-β-induced migration, due mainly to increased PDGF receptor expression. The effect was blocked by downregulation of miR-233, suggesting that the latter has a key role in this process. Surprisingly, recent in vitro data demonstrated that human VSMCs pretreated with dialysis serum displayed significantly less PDGF-induced migration than cells pretreated with normal human serum; this observation suggests that uremic serum may also contain antimigratory factors [7].

### 2.3. Cell Death 

The apoptosis of VSMCs promotes atherogenesis, various aspects of plaque instability, intimal calcification, and medial calcification [58,59]. Vessels from pediatric patients on dialysis display extensive VSMC apoptosis [60], and uremic serum promotes the extensive apoptosis of human VSMCs cultured in vitro [9]. The apoptotic bodies derived from these VSMCs are known to act as nucleating structures for calcium crystals [59], and thus promote vascular calcification. It has therefore been hypothesized that uremic toxins are key contributors (via programmed VSMC death) to the marked atherogenesis and calcification observed in CKD settings.

Apoptosis is a form of programmed cell death that can be induced by two different pathways: the intrinsic pathway is controlled by the mitochondria and by proteins from the BCL2 family, whereas the extrinsic pathway involves death receptors [61]. The balance between death agonists (Bax, Bad and Bak) and antagonists (Bcl-2 and Bcl-xl) from the Bcl-2 protein family has a pivotal role in intrinsic apoptosis. Indeed, PCS significantly increased the percentage of apoptotic VSMCs in vitro through upregulation of Bax and downregulation of Bcl-2 [22]. In vitro, high-Pi-induced human VSMC apoptosis (as evidenced by changes in nuclear shape and phosphatidyl-serine externalization) is associated with the increased expression of pro-apoptotic BCL2 family proteins and caspase-3—suggesting a role for the intrinsic pathway [9,40]. These data were confirmed by the observation that high-Pi-induced downregulation of the anti-apoptotic growth-arrest-specific gene 6 and its receptor Axl induced VSMC apoptosis via increased caspase 3 activation [62]. Interestingly, BAD (a member of the BH3-only pro-apoptotic protein family) is overexpressed in aortic tissues isolated from uremic mouse [63]. In vitro, the upregulation of BAD induced by exposure to urea [63] sensitized VSMCs to the pro-apoptotic effect of oxidized cholesterol (a physiologically relevant inducer of this form of programmed cell death); this observation suggests that the induction of BAD might account (at least in part) for the elevated levels of apoptosis observed in the arteries of uremic patients [60]. However, given that exposure to high Pi also upregulated several death receptors and their ligands (such as soluble TNF receptor 1 and Fas ligand) in VSMCs cultured in vitro, activation of the extrinsic pathway cannot be ruled out [40]. The extrinsic apoptosis pathway induced by the TNF superfamily requires a ligand to bind to its receptor and trigger oligotrimerization of the latter [64,65]. This results in the aggregation of death domain-containing proteins and enables the recruitment of TNF receptor 1-associated death domain protein. The latter binds to Fas-associated death-domain-containing protein and TNF receptor 1-associated protein 2; in turn, these proteins activate procaspase-8 and apoptosis signal-regulating kinase 1 (ASK1), respectively. Tumor necrosis factor alpha (TNFα) induced apoptosis in VSMCs via caspase-8 and 3 activation [66,67] and simultaneous activation of the ASK1-JNK/p38 death signal [68]. In vitro, inhibition of NFkB activation induced TNFα–mediated caspase-3 activity [67], increased the TNF-α-induced expression of p73β [69] (a pro-apoptotic protein that favors the expression of p53 target genes (such as p21) and is involved in plaque progression and rupture), and promoted VSMC apoptosis. Furthermore, the TNF-α induced downregulation of Cx43 induced VSMC apoptosis in vitro [70]. A combination of IFN-γ, TNF-α, and IL-1β induced the apoptosis of cultured human and rat VSMCs through activation of the L-arginine/NOS pathway [71]. Indeed, IL-1β-induced nitric oxide release promoted the apoptosis of rat VSMCs in vitro through upregulation of Fas and a cGMP-independent mechanism [72,73]. Furthermore, inflammatory cytokines (such as TNF-α and IFN-γ) sensitized human VSMCs in vitro to Fas-induced apoptosis by promoting Fas trafficking to the cell surface—a process that involves PI3K, Akt, and Jak-2/Stat1 [74]. Interleukin-6 also induced the apoptosis of human VSMCs in vitro via Stat1 activation in a time-dependent manner [75], and IL-6 partly mediates the TNF-α-induced apoptosis of VSMCs [76]. The RAGE-induced stimulation of NFkB activity promotes VSMC apoptosis. Induction of endoplasmic reticulum (ER) stress by RAGE activation has a key role in this process [77] via the HuR-dependent up-regulation of caspase-9 and Bcl-2 family proteins [78].

### 2.4. Calcification and Osteogenic Differentiation 

Vascular calcification is one of the main features of the vascular remodeling process associated with CKD. This phenomenon is characterized by the accumulation of calcium and phosphate salts within the intimal and medial layers of the vascular wall and within cardiac valves. Intimal calcification generally occurs in association with the development of atherosclerotic plaques, and can be involved in ischemic events. Medial calcification develops preferentially along medial elastic fibers; it leads to vessel stiffness, and favors the development of hypertension, left ventricular hypertrophy, diastolic dysfunction, and heart failure. Vascular calcification is triggered by an imbalance between mineralization inhibitors (magnesium, fetuin-A, matrix Gla protein (MGP) and pyrophosphate (PPi)) and mineralization inducers. In the CKD population, the accumulation of uremic toxins (particularly Pi, IS, AGEs and inflammatory cytokines) is responsible for the high prevalence of vascular calcification. The process is characterized by the conversion of VSMCs into osteochondrogenic cells in response to elevated Pi and calcium levels. During this process, VSMCs downregulate their contractile markers and increase the expression of bone-promoting genes coding for bone morphogenetic protein 2 (BMP2), runt-related transcription factor 2 (RUNX2) and tissue non-specific alkaline phosphatase (TNAP). This phenomenon is associated with the secretion of a procalcific matrix rich in type I collagen and with the production of MMPs 2 and 9, which induces elastin degradation and the subsequent release of procalcific elastin peptides. These pro-calcific conditions also favor the release of VSMC-derived matrix vesicles and apoptotic bodies able to nucleate hydroxyapatite. In turn, the elevated number of Ca/P nanocrystals formed in response to uremia directly promotes the osteochondrogenic conversion of VSMCs, stimulates the production of pro-inflammatory cytokines by resident macrophages, and thereby intensifies mineral deposition. Although hyperphosphatemia is considered to be the main risk factor for the development of vascular calcification in CKD patients [79], there is evidence to suggest that an elevated serum calcium level and an elevated Ca × P product have a pivotal role [80,81]. Indeed, calcium and Pi are not only the strongest inducers of the calcification process but also constitute the main components of hydroxyapatite crystals. Data obtained from cell-based and animal models indicate that elevated extracellular calcium or Pi concentrations induce VSMC calcification independently and synergistically. As discussed above, exposure to high-Pi downregulated miR-143 and miR-145, which consequently upregulated their target genes and the key markers of the VSMC synthetic phenotype KLF4, KLF5 and versican [39]. KLF4 promotes the switch toward an osteogenic VSMC phenotype, and is therefore a key regulator of Pi-induced vascular calcification in rat VSMCs in vitro and in adenine CKD rats in vivo [82]. Interestingly, KLF4 also regulates the transition of VSMCs toward a macrophage-like phenotype [83]. These macrophage-like cells (i) display a reduced ability to clear lipids, dying cells, and necrotic debris; (ii) are known to exacerbate inflammation; and (iii) may promote both atherosclerosis and calcification [3,83]. A Pi-induced increase in KLF4 may therefore amplify both intimal and medial calcification and the development of atherosclerosis. Interestingly, Pi can induce non-polarized macrophages to adopt a phenotype closely resembling that of alternatively activated M2 macrophages [84]. In vitro, these macrophages display an anticalcifying action through the increased availability of extracellular ATP and pyrophosphate, which suggests the existence of a compensatory mechanism that protects tissues from pathologic, hyperphosphatemia-induced calcification. In line with these data, Chinetti-Gbaguidi et al. recently found that macrophages surrounding calcium deposits in human atherosclerotic plaques express the mannose receptor—a marker typically associated with alternatively activated M2 macrophages. However, cathepsin K expression was defective in these macrophages, which suggests that the cells would be unable to resorb calcification in vivo [85].

It is noteworthy that exposure to TNF-α amplifies the Pi-induced osteogenic transition of VSMCs and their subsequent calcification [85]. In particular, TNF-α activation of the NFkB pathway induced the expression of the osteogenic transcription factor MSX2, which in turn increases TNAP activity both in vitro and in animal models [86,87]. In VSMCs, TNF-α activation of the NFkB pathway also inhibited the expression of ankylosis protein homolog (a transmembrane protein that exports PPi to the extracellular space, resulting in reduced PPi release) [88]. Furthermore, TNF-α activation of the PERK-eIF2a-ATF4-CHOP axis in cultured VSMCs favored Pi uptake and subsequent calcification through increased expression of the sodium-dependent phosphate transporter (Pit-1) [89]. Likewise, TNF-α-induced activation of the PERK-eIF2α-ATF4-CHOP axis promoted ER-stress-dependent calcification of VSMCs in 5/6 nephrectomized ApoE^−/−^ mice [89]. Moreover, TNF-α–induced downregulation of vascular Klotho expression might indirectly promote vascular calcification [90,91,92]. The previously described amplification by TNF-α of Pi-induced VSMC apoptosis also promotes calcification [93]. Interleukin-6 is also a key inducer of the osteogenic transition and mineralization in VSMCs [94]. In vitro, the IL-6-induced expression of heat shock protein 70 abolished MGP’s inhibitory effect on the activity of BMP, which promoted VSMC calcification [95]. Interestingly, IL-6 was reported to induce BMP2, TNAP and osteopontin (OPN) expression and the subsequent calcification of VSMCs cultured in vitro under non-calcific conditions (i.e., in the absence of osteogenic medium) [96]. Interleukin-6 is a strong inducer of receptor activator of nuclear factor kappa-B ligand (RANKL) and, conversely, is modulated by RANKL [97,98]. Neutralization of IL-6 reduced RANKL-induced OPN, Runx2, and BMP2 mRNA expression and inhibited the RANKL-dependent amplification of Pi-induced VSMC calcification in vitro [98]. Recent data show that the elevated levels of TNF-α in uremic serum promoted the osteochondrogenic VSMC transition and then calcification via the ERK- and AP1/c-fos-mediated induction of IL-6 [76]. These findings suggest that most of the well-known effects of TNF-α on VSMC calcification depend on IL-6’s pro-apoptotic, pro-oxidative, and pro-osteogenic actions [94].

Oxidative stress and AGEs are associated with extensive coronary artery calcification in hemodialysis patients [99]. In vitro, the binding of AGEs to RAGE accelerated the Pi-induced osteogenic transition in VSMCs and subsequent calcification through P38/MAPK and Wnt/β catenin signaling [100,101,102]. Activation of RAGE repressed the expression of smooth muscle cell marker genes by inhibiting the transactivating function of myocardin [103]. This effect was associated with a concomitant increase in osteogenic VSMC differentiation through Notch/Msx2 induction [103]. Moreover, in vitro results suggest that induction of NADPH oxidase activity by AGEs is involved in RAGE-dependent calcification [104,105]. In the vasculature of CKD mice, RAGE-induced oxidative stress promoted osteogenic VSMC differentiation and subsequent medial calcification [104]. In experiments in ApoE^−/−^ mice, RAGE-induced NADPH oxidase promoted advanced calcification within atherosclerotic lesions in the innominate artery, and medial calcification in the aortic arch by increasing the osteogenic transition [105]. In diabetic rats, AGE inhibitors prevented vascular calcification [106]. In this model, diabetes-accelerated calcification was also prevented by antioxidants.

In CKD patients, serum IS levels were positively correlated with aortic calcification [107], suggesting that IS may act as a procalcific toxin. Indeed, in Dahl salt-sensitive hypertensive rats, IS administration induced aortic wall thickening and aortic calcification, with the expression of osteoblast-specific protein [108]. In vitro, IS increased Pi-induced VSMC calcification and osteogenic transition by increasing Pit-1 expression [109], oxidative stress [110] and senescence [20]. Indoxyl sulfate also increased methylation and subsequent transcriptional suppression of the α-Klotho gene, which promoted vascular calcification in VSMCs cultured in vitro and in 5/6-nephrectomized Sprague Dawley rats [108].

Over the last few decades, increasing attention had been paid to the role of the FGF-23/Klotho axis in the development of vascular calcification. The fact that FGF23-knockout mice have a vascular calcification phenotype [111] was initially suggestive of direct, FGF-23-mediated protection against vascular calcification. Accordingly, exposure to exogenous FGF-23 was reported to protect against vascular calcification in cultured VSMCs [112], and α-Klotho knockdown abolished this FGF-23-mediated protection in vitro [91]. Furthermore, complete neutralization of FGF-23 in CKD rats accelerated vascular calcification and increased mortality [113]. In line with these data, transgenic uremic mice overexpressing α-Klotho exhibited less calcification than wild-type uremic mice [92], and exposure to soluble α-Klotho suppressed the high-Pi-induced osteogenic VSMC transition and subsequent mineralization in vitro [92]—suggesting the existence of vascular resistance to FGF-23 in CKD because of a concomitant vascular α-Klotho deficiency. However, contradictory data showed that exposure to FGF-23 enhanced Pi-induced vascular calcification by promoting osteoblastic transdifferentiation in aortic rings from uremic rats [114]. More confusingly still, another study found that exposure to FGF-23 had no effect on Pi-induced calcification of VSMCs, despite the presence of α-Klotho [115,116]. Therefore, additional studies are needed to elucidate the precise role of FGF-23 in the pathogenesis of vascular calcification in CKD.

According to recent data, urea may display direct pro-calcific effects on VSMCs through a nonenzymatic posttranslational modification named protein carbamylation [117]. The term “carbamylation” refers to the covalent binding of isocyanic acid to α-amino groups of free amino acids or N-terminus of proteins, and to ε-amino groups of lysine residues [118]. Carbamylation is a physiological process of molecular aging in mammal species, which modifies proteins charge with consequences for their structure, function, and interactions. In a recent study, Mori and colleagues reported that urea-induced protein carbamylation may play a key role in VSMC calcification [117]. In this report the authors demonstrated that urea-induced carbamylation of ATP synthase subunits α and β induced mitochondria derived oxidative stress, which suppressed the expression of ENPP1, a key enzyme that generates the calcification inhibitor PPi. The downregulation of ENPP1 observed after urea-induced protein carbamylation significantly amplified the calcification of VSMC cultured in vitro, an effect that was blocked by addition of the carbamylation inhibitor glycylglycine. Confirming these observations, feeding heminephrectomized SD rats with a diet containing 20% urea significantly increased the carbamylation of ATP synthase subunits α and β as compared to heminephrectomized animals fed a control diet. In vitro, aortic rings from heminephrectomized rats fed a diet containing 20% urea showed increased vascular calcification and reduced ENPP1 expression after exposure to a procalcific medium as compared to aortic rings isolated from heminephractomized rats fed a control diet. Protein carbamylation was also enhanced in the tunica media of a patient with end-stage renal disease and colocalized with ATP synthase subunit α. Taken together, these data suggest that urea-induced carbamylation of the ATP synthase subunit α in human might promote vascular calcification development through downregulation of ENPP1 and subsequent inhibition of PPi formation. Together, these findings uncover a previously unrecognized aspect of uremia-related vascular calcification in which urea-induced carbamylation plays a central role.

### 2.5. Senescence 

Senescence is a protective mechanism engaged in response to excessive cellular stress. It induces a permanent cell cycle arrest, and prevents the transmission of defects to the next generation. Senescence can occur after repeated cell division or can be induced by a variety of stresses, including mitochondrial deterioration, DNA damage and oxidative stress—all processes to which VSMCs are exposed within the uremic milieu. It is now acknowledged that VSMC senescence impairs vascular repair and is accompanied by fibroblast invasion, vascular calcification, and the development of atherosclerosis [3]. Senescent VSMCs are large, flattened and star-shaped. They have short telomeres, accumulate prelamin A, and contain elevated levels of senescence-associated markers (including senescence-associated β-galactosidase (SAβG) and cell cycle regulators (such as p16, p21, p53, and pRb) [119]. Senescent cells are known to have a specific, senescence-associated secretory phenotype (SASP) that affects the surrounding tissue and alters local homeostasis. In an elegant study, Gardner and colleagues observed that senescent VSMCs secrete many cytokines (including IL-6 and -8) in an IL-1α-dependent manner [120]. In the latter study, SAPS VSMCs secreted abnormally high levels of MMP9, downregulated collagen, and upregulated inflammasome components. Secretion of IL-1α by senescent human VSMCs was found to drive the SASP in an autocrine manner [120]. Interestingly, SAPS VSMCs primed adjacent control VSMCs to adopt a proinflammatory state, and promoted the chemotaxis of mononuclear cells both in vitro and in vivo—suggesting that VSMCs may actively contribute to the chronic inflammation associated with atherosclerosis [120]. Taken as a whole, these data highlight the existence of a vicious circle through which VSMC dysfunction accentuates VSMC dysfunction. In accordance with these observations, unstable, mature plaques show clear evidence of VSMC senescence [119,120], and restenosed neointimas also contain senescent VSMCs [121]. It is noteworthy that vascular tissues from CKD animals contain elevated levels of SaβG [122,123], suggesting that uremia promotes CVD by increasing VSMC senescence.

Hyperphosphatemia has been linked to a premature aging phenotype in Klotho-deficient mice and in FGF23 KO mice, whereas the dietary restriction of Pi reversed the aging phenotype in the latter animal model [124,125,126]. These observations suggest that exposure to high Pi concentrations can induce cell senescence [122]. Indeed, high Pi concentrations reportedly induced the senescence of human VSMCs cultured in vitro [122,123]. This phenomenon partly depends on integrin-linked kinase’s (ILK) activation of the aging-related signaling pathway involving IGF-1/AKT/FoxO, with a subsequent increase in ROS production [123]. These results were confirmed by the observation that rat aortas cultured ex vivo in high-Pi medium expressed high levels of ILK and upregulated the transcription of the senescence genes p53 and p16 [123]. The same observation has been made in nephrectomized rats fed a hyperphosphatemic diet [123]. As discussed previously, Rahabi-Layachi et al. recently reported decreased VSMCs proliferation after cell cycle arrest in response to high Pi in human VSMCs cultured in vitro [40]. This effect was associated with low pRb phosphorylation and high expression of p21 (a cell cycle inhibitor involved in vascular senescence [127]). Therefore, the possibility that the anti-proliferative effects observed by Rahabi Layachi [40] and others [39] in response to high-Pi may represent an early event of VSMC senescence cannot be ruled out. Further studies will be needed to clarify this concern.

Recent, microarray analysis of senescent VSMCs revealed the differential regulation of genes associated with vascular calcification, including MGP, BMP2, osteoprotegerin, and OPN [128]—suggesting that VSMC senescence may impact the development of vascular calcification. In accordance with these data, Pi-induced VSMC senescence was associated with increased calcification in vitro through the downregulation of SIRT1 expression and subsequent p21 activation [122]. Lastly, elevated SAβG activity had been reported in calcified vessels from rats with adenine-induced renal failure and hyperphosphatemia [122].

Oxidative stress is one of the most physiologically relevant triggers of cell senescence under pathological conditions. Indoxyl-sulfate-induced-oxidative stress promoted the senescence of VSMCs [20] via the upregulation of p53, p21 and prelamin A, and downregulation of FACE1 (a metalloproteinase involved in the maturation of prelamin A). In the latter study, administration of IS to Dahl salt-resistant hypertensive rats markedly increased the expression of senescence biomarkers (including SAβG, p53, p21, and prelamin A) and oxidative stress biomarkers (such as 8-hydroxyl-2′-deoxyguanosine, and malondialdehyde) in the cells embedded in the calcification area of the arcuate aorta [20].

## 3. Conclusions

Although our understanding of the molecular mechanisms underlying the induction of VSMC dysfunction in CKD has improved considerably, it is not yet possible to prevent or cure vascular remodeling (i.e., intimal hyperplasia, accelerated atherosclerosis, excessive vascular calcification, and vascular stiffness) in this context. This is notably due to the complexity and multifactorial nature of CKD-induced VSMC dysfunction (i.e., mineral, metabolic and hemodynamic disturbances). The present review suggests that targeting uremic toxins may be a promising strategy for preventing the development or progression of VSMC dysfunction and subsequent vascular remodeling in patients with CKD. Accordingly, the administration of AST-120 (which decreases serum levels of IS) to pre-dialysis CKD patients reduced carotid intima-media thickness and pulse wave velocity [129], prevented the development of left ventricular concentric change [130], and decreased aortic calcification [131]. Furthermore, Pi-binders (such as sevelamer hydrochloride and lanthanum carbonate) decreased the development and/or progression of vascular calcification [132,133] and atherosclerosis [134] in animal models of CKD. It appears that the oxidative burden promoted by retained uremic solutes is one of the main causes of VSMC dysfunction. Therefore, it was initially thought that antioxidant therapy might be beneficial in reducing oxidative stress, lowering uremic cardiovascular toxicity, and improving survival. However, in a recent meta-analysis investigating the use of antioxidants for people with CKD, antioxidant therapies showed no clear overall effect on cardiovascular mortality, all-cause mortality, cardiovascular disease, coronary heart disease, cerebrovascular disease or peripheral vascular disease compared with placebo [135]. Nevertheless, a subgroup analyses raised the possibility of cardiovascular and coronary benefit from antioxidant therapy in the dialysis population. Indeed, two studies undertaken in populations of participants undergoing haemodialysis found that cardiovascular mortality, cardiovascular events and peripheral vascular disease were substantially reduced in antioxidant therapy arm participants compared with those who received placebo [136,137,138]. However, the small size and generally suboptimal quality of the included studies meant that the reliability of this result remains open to question. The possibility of a cardiovascular benefit in dialysis patients with antioxidant therapies needs to be tested in sufficiently powered studies, specifically involving this population. Dialysis therapy reduces the concentration of oxidized substrates and may thus also improve the redox balance. In this context, the use of high cut-off dialysis membranes to remove inflammatory cytokines and AGEs may reduce VSMC dysfunction and therefore slow the progression of uremic vascular remodeling [139]. The improvement of dialysis processes (which can contribute *per se* to a pro-inflammatory, pro-oxidative state) may also reduce VSMC dysfunction. Kidney transplantation slows the progression of coronary artery calcification [140], and patients with end-stage renal disease display a reduction in overall arterial stiffness after kidney transplantation [141]. However, coronary artery calcification continues to progress (albeit more slowly) after kidney transplantation, and vascular calcification is more severe in long-term kidney transplant recipients than in matched patients with stage 5/5D CKD [142]. Vascular calcification increases with time since transplantation, and is partly influenced by the patient’s history of dialysis. These findings suggest that certain dysfunctions are irreversible and/or self-sustaining, and justify the search for preventive treatments. More intense investigation (including clinical research) will be needed to develop novel strategies for the management of VSMC dysfunction in a uremic setting.

## Figures and Tables

**Table 1:** **Summary of the mechanisms by which uremic toxins impact VSMC function**. AGE: advanced glycation end product, ANKH: ankylosis protein homolog, FAK : focal adhesion kinase, FasL: fas ligand, ER: endoplasmic reticulum, IL-1β : interleukin 1β, IL-6 : interleukin 6, IS: indoxyl-sulfate, KLF4: Kruppel-like factor 4, KLF5: Kruppel-like factor 5, MMP: matrix metalloproteinase, NO: nitric oxide, NQO1: NAD(P)H:quinone oxidoreductase 1, PCS: paracresyl-sulfate, PDGF: platelet-derived growth factor, PDGFB-R: PDGF-β receptor, Pit-1: sodium-dependent phosphate cotransporter type III, PPi: pyrophosphate, RAGE: receptor for advanced glycation end products, RANKL: receptor activator of nuclear factor kappa-B ligand, pRb: retinoblastoma protein, ROS: reactive oxygen species, SAβG: senescence-associated β-galactosidase, TGF-β: transforming growth factor beta. TNAP: tissue non-specific alkaline phosphatase, TNF-α: tumor-necrosis factor alpha, TRAIL: tumor-necrosis-factor-related apoptosis-inducing ligand. ND: No data.

Uremic Toxins	Proliferation		Migration		Apoptosis		Calcification		Senescence	
	Effect	Mechanisms of Action	Effect	Mechanisms of Action	Effect	Mechanisms of Action	Effect	Mechanisms of Action	Effect	Mechanisms of Action
**phosphate**	−	G1/S Blocade ↓Cyclin E, CDK2 ↓Phospho pRb ↑p15, p21, p27	+	↑miR223 ↓miR143, miR145 ↑KLF4, KLF5 ↑versican ↑PDGFR	+	↑Caspase 3 ↑BAD ↑FasL ↑TNF-R1 ↓Gas6	+	↑Osteogenic transition ↑MMP9 ↑Apoptotis/senescence ↑Procalcific MVs ↓miR143/miR145 ↑KLF4/↑miR223	+	↑ILK ↑ROS ↑p53, p16, p21 ↑G1/S Blocade ↓Phospho Rb ↑SAβG
**IS**	Acute +	↑ROS ↑PDGFB-R ↑Cyclin D1, P21 ↓p53 ↑GLUT1	+	↑ROS ↑PDGFB-R	ND		+	↑Osteogenic transition ↑Pit-1 ↓klotho	+	↑ROS ↑p21, p53 ↑Prelamin A ↓FACE1 ↑SAβG
	Chronic −	↑ROS ↑P21, P27								
**PCS**	To be clarified		+	↑MMP2/9 ↓TIMP1/2	+	↑Bax ↓Bcl2	ND		ND	
**AGEs**	+	↑ROS ↑Inflammation	+	↑ROS ↑MMP2/9 ↑TGF-β ↑Lipocalin 2 ↑PDGF ↑Inflammation	+	↑ER stress ↑BAD ↑Caspase 9	+	↑ROS ↑Osteogenic transition	ND	
**TNF-α**	+	↑ROS ↑TRAIL ↑IL-6 ↑PDGFβ	+	↑MMP2/9 ↑TRAIL ↑NQO1 ↑IL-6	+	↑Caspase 3/8 ↑p73β ↓Cx43 ↑Fas	+	↑Osteogenic transition ↓ANKH = PPi↓ ↑Pit-1 ↑ER stress ↓Gas6 = apoptosis↑ ↓Klotho ↑IL-6	ND	
**IL-6**	+	Mediates TNF-α effects	+	↑Actin polymerization ↑Phospho FAK/paxillin Mediates TNF-α effects	+	↑Stat1 Mediates TNF-α effects	+	↑HSP70 ↑RANKL Mediates TNF-α effects	ND	
**IL-1β**	+	↑PDGFβ ↑P2Y_2_R ↑RAGE ↑HMGB1	+	↑MMP2 ↑P2Y_2_R	+	↑Fas ↑NO	+	Induction of TNAP	ND	
**FGF-23**	ND		No influence		ND		To be clarified		ND	
